# Clinical factors associated with outcomes among patients receiving second-line therapy for advanced NSCLC

**DOI:** 10.2340/1651-226X.2026.46269

**Published:** 2026-10-28

**Authors:** Vasileios Karagiannis, Levi Byskata, Nelli-Sofia Nåhls, Eero Sihvo, Antti Jekunen, Mikael Johansson, Heidi Andersén

**Affiliations:** a Department of Oncology, Vaasa Central Hospital, The Wellbeing Services County of Ostrobothnia, Vaasa, Finland; b Faculty of Medicine, University of Helsinki, Helsinki, Finland; c Department of Surgery, Hospital Nova, Jyväskylä, Finland; d Department of Clinical Oncology, University of Turku, Turku, Finland; e Department of Diagnostics and Intervention, Oncology, Umeå University, Umeå, Sweden; f Faculty of Medicine and Health Technology, Tampere University, Tampere, Finland

## Introduction

Despite advances in non-small cell lung cancer (NSCLC) treatment, many patients progress to advanced disease, and most subsequently develop progression after first-line (1L) therapy, requiring consideration of further systemic treatment [1]. A substantial proportion does not receive second-line (2L) systemic therapy because of clinical deterioration, toxicity or declining performance status (PS) after 1L treatment [2, 3]. Among patients who remain eligible, 2L treatment decisions require careful estimation of both expected benefit and treatment burden, taking into account PS, comorbidity, prior treatment exposure and toxicity, molecular profile and available later-line options [1, 4].

Evidence on clinical predictors of 2L outcomes remains heterogeneous; many studies are treatment-specific or pre-immunotherapy, with limited integration of the preceding treatment course, toxicity, PS, frailty and radiologic outcomes [3, 5–9].

This study aimed to describe outcomes and prognostic associations among patients with advanced or metastatic NSCLC who received second-line systemic therapy in routine clinical practice.

## Materials and methods

### Study design and setting

We conducted a population-based retrospective cohort study at Vaasa Central Hospital, Finland, including all patients diagnosed with histologically confirmed NSCLC between 1 January 2016 and 31 December 2022. The present analysis included patients who received two or more systemic treatment lines for metastatic NSCLC within the hospital network. All 106 patients had metastatic NSCLC at the time of second-line treatment and had received at least two systemic treatment lines for disease progression. The stage at initial diagnosis was not necessarily stage IV. Some patients developed metastatic recurrence after earlier-stage disease. Palliative radiotherapy before 2L was not an exclusion criterion and was evaluated as a covariate (Supplementary Tables 3, 6 and 8). Patients treated exclusively outside the hospital network were excluded. Patients who received only neoadjuvant or adjuvant systemic therapy were excluded (Supplementary Figure 1).

A new treatment line was defined as the initiation of a systemic regimen involving a new agent or drug class. Dose reductions, treatment breaks and maintenance continuation were not counted as new lines.

### Data sources and variables

Clinical variables were obtained from existing datasets [10, 11] and supplemented by structured medical record review. Variables included demographics, histology and stage, driver mutation status, treatment characteristics, comorbidity (Charlson Comorbidity Index [CCI]), Clinical Frailty Scale (CFS), PS and adverse events (AEs) graded by the Common Terminology Criteria for Adverse Events (CTCAE) v5.0. Radiologic response and progression were assessed according to the Response Evaluation Criteria in Solid Tumours (RECIST) 1.1 by two radiologists.

### Statistical analysis

Analyses were performed using IBM SPSS Statistics version 28.0 (IBM, Armonk, NY, USA). Time-to-event outcomes were analysed using Kaplan–Meier methods and Cox proportional hazards regression and radiologic response using logistic regression. Candidate variables required clinical relevance, less than 10% missing data and univariable *p* < 0.05. Driver mutation status was considered as a candidate variable for the multivariable overall survival (OS) model but was excluded due to model instability related to the small driver mutation-positive subgroup (*n* = 16).

Detailed crude, adjusted and sensitivity analyses are provided in the Supplementary Appendix. No a priori power calculation was performed, and the study was not designed to compare treatment regimens. No imputation was performed; models were fitted to complete cases, and the analysed n is reported with each estimate. The proportional hazards assumption was assessed graphically with log-minus-log plots; no clear departures were observed.

### Ethical approval

This study was conducted in accordance with the Declaration of Helsinki. The detailed NSCLC cohort was originally established under prospective permissions from the National Institute for Health and Welfare of Finland (THL/143/5.05.00/2015; THL 1349/505.00/215) and Statistics Finland (TK53–1410–15). Ethical approval for the retrospective analysis was granted by the hospital board (OPVH_2022_105JYL). All patient data were handled within the hospital network, and identifying information was removed prior to analysis to ensure anonymity.

## Results

Of 458 patients with histologically confirmed NSCLC identified during 2016–2022, 210 received systemic therapy for metastatic disease. The analysis included 106 patients who received at least two systemic treatment lines; 24.5% had PS 2 before 1L and 15.1% had a driver mutation (Table 1).

**Table 1:** Baseline demographic and clinical characteristics of patients receiving two or more lines of systemic therapy for advanced NSCLC (n = 106).

Characteristics	Total (*n* = 106)	Male (*n* = 68)	Female (*n* = 38)
Median age (years, IQR)			
At 1L start	71 (63–76)	72 (64–76)	71 (62–76)
At 2L start	72 (65–77)	73 (65–77)	72 (63–78)
Median BMI (IQR), before 1L	24.3 (22.7–27.5)	24.7 (23.0–27.7)	24.2 (21.1–27.3)
Smoking history before 1L *n* (%)			
Current	18 (16.9)	14 (20.6)	4 (10.5)
Former	45 (42.5)	29 (42.6)	16 (42.1)
Never	43 (40.6)	25 (36.8)	18 (47.4)
Median pack-years (IQR)	40 (15–50)	40 (20–50)	26 (0–40)
Known asbestos exposure *n* (%)			
Yes	20 (18.9)	19 (27.9)	1 (2.6)
No	86 (81.1)	49 (72.1)	37 (97.4)
Performance status before 1L *n* (%)			
0–1	80 (75.5)	49 (72.1)	31 (81.6)
2	26 (24.5)	19 (27.9)	7 (18.4)
Performance status before 2L *n* (%)			
0–1	68 (64.2)	42 (61.8)	26 (68.4)
2	37 (34.9)	25 (36.8)	12 (31.6)
3	1 (0.9)	1 (1.5)	0 (0)
Charlson Comorbidity Index, before 1L *n* (%)			
0	40 (37.7)	23 (33.8)	17 (44.7)
1–2	49 (46.2)	33 (48.5)	16 (42.1)
3–4	14 (13.2)	9 (13.2)	5 (13.2)
≥ 5	3 (2.8)	3 (4.4)	0 (0)
Clinical Frailty Scale before 1L *n* (%)			
Robust (1–2)	44 (41.5)	28 (41.2)	16 (42.1)
Pre-frail (3–4)	47 (44.3)	33 (48.5)	14 (36.8)
Frail (≥ 5)	15 (14.2)	7 (10.3)	8 (21.1)
Histological subtype *n* (%)			
Adenocarcinoma	73 (68.9)	44 (64.7)	29 (76.3)
Squamous cell carcinoma	28 (26.4)	21 (30.9)	7 (18.4)
Other or unspecified	5 (4.7)	3 (4.4)	2 (5.3)
Driver mutation positivity *n* (%)			
Yes	16 (15.1)	7 (10.3)	9 (23.7)
No	76 (71.7)	50 (73.5)	26 (68.4)
Test not done	14 (13.2)	11 (16.2)	3 (7.9)
Primary TNM stage *n* (%)			
I–II	11 (10.4)	7 (10.3)	4 (10.6)
III	26 (24.5)	19 (27.9)	7 (18.4)
IV	69 (65.1)	42 (61.8)	27 (71.1)
Median eGFR (IQR)			
Before 1L	88 (74–95)	87 (73–94)	92 (77–96)
Before 2L	83 (69–89)	83 (73–88)	83 (64–92)
Median CRP before 2L (IQR)	11 (3–31)	12 (5–31)	7 (2–34)
Median plasma albumin before 2L (IQR)	34 (31–36)	34 (31–37)	34 (29–36)

Data are presented as median (IQR) for continuous variables and *n* (%) for categorical variables. IQR: interquartile range; BMI: body mass index; PS: performance status; CCI: Charlson Comorbidity Index; CFS: Clinical Frailty Scale; TNM: Tumour-Node-Metastasis; eGFR: estimated glomerular filtration rate; CRP: C-reactive protein; 1L: first-line; 2L: second-line. Primary TNM stage refers to the stage at initial diagnosis; all patients had metastatic disease at 2L initiation.

### Second-line effectiveness

During 2L therapy (*n* = 106), best radiologic response was a complete response (CR) in 0.9%, a partial response (PR) in 8.5%, a stable disease (SD) in 53.8% and a progressive disease (PD) in 36.8% (Figure 1). Median progression-free survival (PFS) during 2L was 5.7 months (95% confidence interval [CI] 2.5–11.5), and median OS from 2L start was 12.4 months (95% CI 6.7–22.7) (Supplementary Table 1).

**Figure 1:**
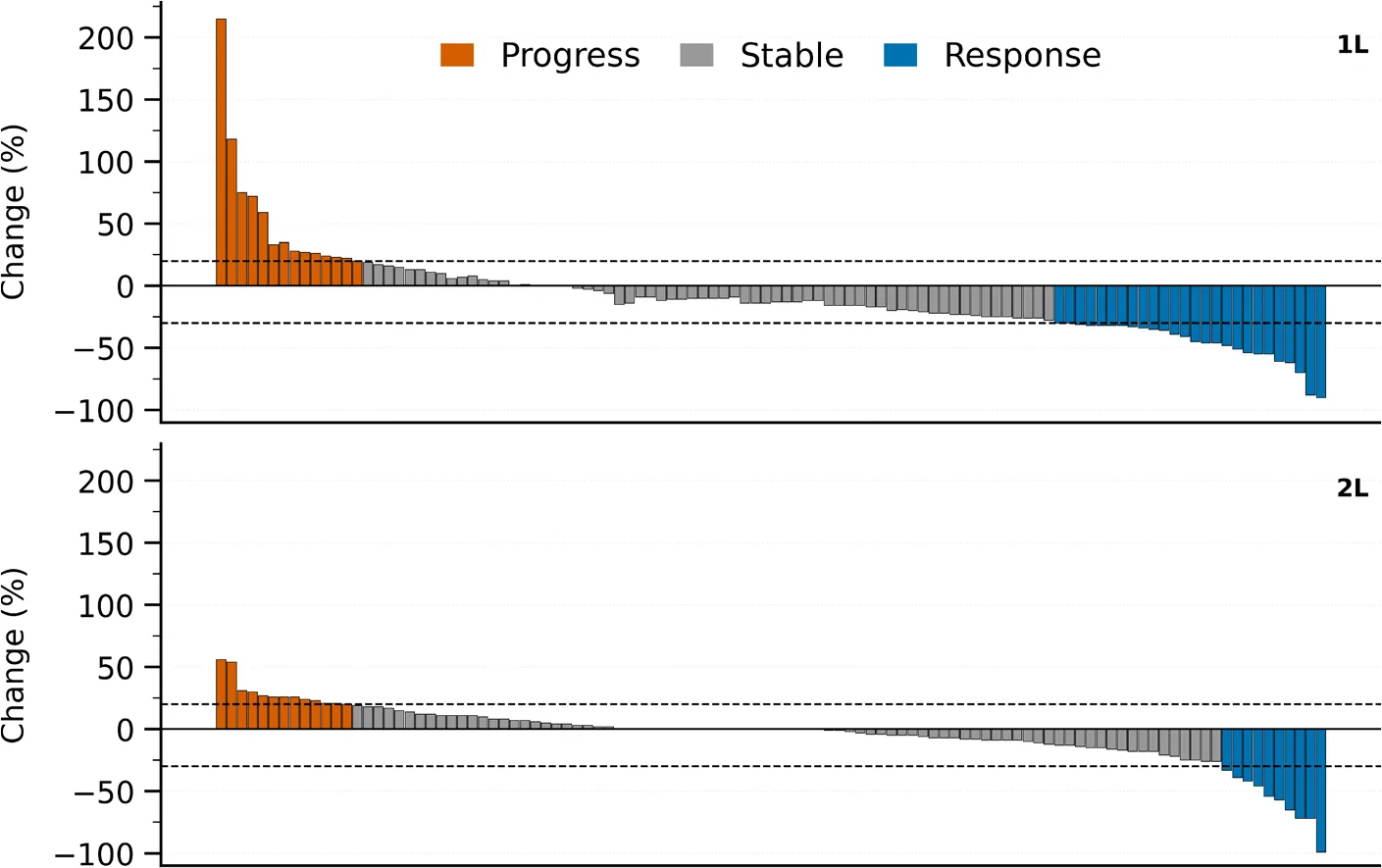
Waterfall plots of best per cent change in tumour burden during first-line and second-line therapy (*n* = 106). Patients are ordered by second-line response (left to right: greatest increase to greatest reduction). Dashed horizontal lines indicate –30% (partial response threshold) and +20% (progressive disease threshold), with a solid line at 0%.

### Adverse events in second-line treatment

AEs were common, affecting 89.6% of patients; 48.1% experienced grade ≥3 AEs. The most frequent AEs were fatigue and gastrointestinal events, with severe AEs mainly being infections, haematologic and gastrointestinal toxicities (Supplementary Table 2).

### Prognostic factors

A comprehensive set of clinical, demographic and treatment-related variables were evaluated as potential prognostic factors for 2L outcomes using a structured selection process based on clinical relevance and data completeness (Supplementary Tables 3–9). In exploratory adjusted models, the strongest and most consistent prognostic factors for worse 2L outcomes were shorter 1L PFS, CTCAE grade 3–4 toxicity in 1L and PS 2. Driver mutation positivity was associated with more favourable crude survival and PFS outcomes, whereas robust frailty status was independently associated with lower odds of PD. Overall, 54.7% (58/106) survived ≥12 months from 2L treatment initiation. Twelve-month OS was 69.1% without versus 39.2% with grade 3–4 1L toxicity, and 68.4, 60.7 and 30.8% for PS 0, 1 and 2, respectively (Supplementary Table 5).

## Discussion

Second-line systemic therapy for advanced NSCLC most commonly resulted in disease stabilisation, with a median PFS of 5.7 months and median OS of 12.4 months from 2L treatment initiation. Nearly half of patients experienced grade ≥3 AEs during 2L therapy. Approximately half of patients receiving systemic therapy for metastatic disease proceeded to 2L, consistent with previous real-world data [2, 3].

The predominance of SD rather than objective response during 2L therapy is consistent with contemporary real-world analyses of subsequent-line treatment in advanced NSCLC, where disease control rather than tumour shrinkage often represents the main observed benefit [3, 12]. Median OS from 2L treatment initiation was slightly more favourable than in several contemporary real-world reports, possibly reflecting differences in patient selection, molecular characteristics, smoking history and treatment patterns across cohorts [3]. The driver mutation-positive subgroup, although small, showed more favourable crude survival, in line with the prolonged survival after relapse reported for epidermal growth factor receptor (EGFR)-mutated disease [13] and with the rapid uptake of molecular testing and targeted therapy in Nordic populations over the same period [14].

The prognostic factors identified in this study are consistent with previous primary studies evaluating second-line outcomes. Chemotherapy- and EGFR tyrosine kinase inhibitor (TKI)-era analyses identified PS, baseline clinical condition and response or disease control during prior therapy as relevant predictors of later-line outcomes [2, 6, 15, 16]. More recent immunotherapy-era cohorts have similarly linked outcomes to treatment line, prior disease control, clinical condition and treatment tolerability [5, 7–9, 17, 18]. In our cohort, PS assessed before 1L was more strongly associated with 2L outcomes than PS immediately before 2L, and robust frailty status before 1L was associated with lower odds of PD, suggesting that baseline physiological reserve retains prognostic relevance across treatment lines. These observations are exploratory. Together with 1L PFS and 1L toxicity, they indicate that routinely recorded information from the 1L course, rather than 2L baseline characteristics alone, describes the trajectory on which 2L outcomes depend.

Selection and survivorship bias are inherent because analyses were restricted to patients who remained eligible for 2L therapy. The findings apply only to patients who initiated second-line treatment. The retrospective single-centre design, modest sample size, low number of objective responses and missing data limit precision and generalisability. Driver-positive patients were retained to preserve the real-world cohort but were too few for reliable treatment-specific interpretation. Attribution of non-specific symptoms such as fatigue to treatment rather than disease progression also remains uncertain. Treatment practices and molecular diagnostics evolved during the 2016–2022 study period, potentially introducing clinical heterogeneity. Toxicity was clinician-graded; patient-reported outcomes, increasingly used in NSCLC studies [19], were not available. Findings should therefore be interpreted as prognostic associations and require validation in larger multicentre cohorts.

## Conclusion

Outcomes after 2L treatment initiation were associated with the preceding 1L disease course, particularly duration of disease control and severe toxicity. The associations should be interpreted as markers of overall disease and treatment trajectory rather than predictors of 2L treatment benefit.

## Supplementary Material



## Data Availability

The data underlying this study are not publicly available due to patient confidentiality and institutional data protection policies but may be available from the corresponding author on reasonable request and with appropriate institutional approvals.
