# Supplementary material for: Clinical factors associated with outcomes among patients receiving second-line therapy for advanced NSCLC
Source: Acta Oncol. 2026 Oct 28;65:46269. doi: 10.2340/1651-226X.2026.46269 (PMC13723031; doi:10.2340/1651-226X.2026.46269)
Supplement: Supplementary file 1 [file AO-65-46269-s1.pdf]

**Supplementary material has been published as submitted. It has not been copyedited, or typeset by Acta Oncologica**

Supplementary appendix.

Title: Clinical Factors Associated with Outcomes Among Patients Receiving Second-Line Therapy for Advanced NSCLC

Authors: Vasileios Karagiannis*, Levi Byskata*, Nelli-Sofia Nåhls, Eero Sihvo, Antti Jekunen, Mikael Johansson, Heidi Andersén

* contributed equally to this work and share first authorship

Supplementary Figure 1. Flow of patients with non-small cell lung cancer (NSCLC) in Ostrobothnia, Finland (2016–2022)

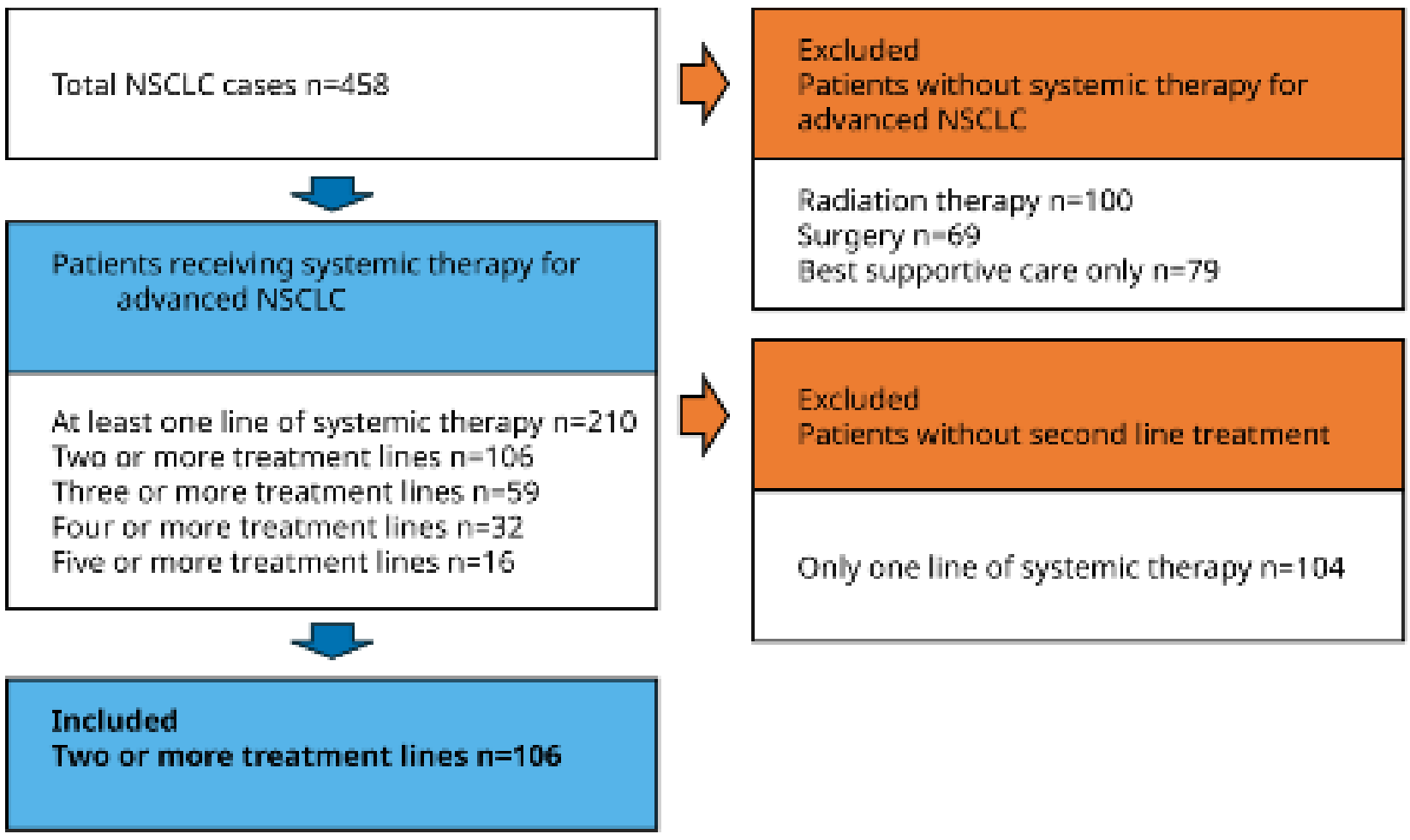

Supplementary tables

Supplementary Table 1. Response rates and survival outcomes by treatment line and cohort.

| | **First line, all patients (n=185)** | **First line, ≥2 lines (n=106)** | **Second line, ≥2 lines (n=106)** | **One line only (n=79)** |
|---|---|---|---|---|
| **Best radiologic response** | | | | |
| **Complete response, n (%)** | 6 (3.2) | 4 (3.8) | 1 (0.9) | 2 (2.5) |
| **Partial response, n (%)** | 32 (17.3) | 20 (18.9) | 9 (8.5) | 12 (15.2) |
| **Stable disease, n (%)** | 53 (28.6) | 38 (35.8) | 57 (53.8) | 15 (19.0) |
| **Progressive disease, n (%)** | 94 (50.8) | 44 (41.5) | 39 (36.8) | 50 (63.3) |
| **Progression-Free Survival** | | | | |
| **Median PFS (95% CI) (months)** | 4.9 (2.3-10.0) | 6.4 (3.3-10.5) | 5.7 (2.5-11.5) | 3.8 (1.2-8.7) |
| **Overall Survival** | | | | |
| **Median OS (95% CI) (months)** | 12.6 (6.7-29.3) | 24.9 (11.3-33.8) | 12.4 (6.7-22.7) | 6.7 (3.5-12.3) |

First-line outcomes are shown for 185 of 210 patients who received systemic therapy; 25 patients were excluded because of missing outcome data. The one-line column therefore includes 79 evaluable patients who did not proceed beyond first-line treatment, whereas Supplementary Figure 1 shows all 104 patients who received only one systemic treatment line. The ≥2-line cohort includes patients who subsequently received second-line therapy, whereas the one-line cohort includes patients who did not proceed beyond first-line treatment. Survival estimates were calculated using Kaplan-Meier analysis and are reported with 95% CIs for the median. Radiologic response was assessed according to RECIST 1.1. Abbreviations: CI, confidence interval; OS, overall survival; PFS, progression-free survival; RECIST, Response Evaluation Criteria in Solid Tumors.

Supplementary Table 2. Adverse events during second-line therapy graded by Common Terminology Criteria for Adverse Events (CTCAE) v5.0

| **n=106 patients** | **Any grade (% of patients)** | **Grade ≥3 (% of patients)** |
|---|---|---|
| **Any adverse events** | 95 (89.6%) | 51 (48.1%) |
| **Fatigue** | 39 (36.8%) | 6 (5.7%) |
| **Nausea** | 19 (17.9%) | 2 (1.9%) |
| **Pain** | 14 (13.2%) | 1 (0.9%) |
| **Infection** | 28 (26.4%) | 23 (21.7%) |
| **Respiratory** | 19 (17.9%) | 8 (7.5%) |
| **Gastrointestinal** | 37 (34.9%) | 12 (11.3%) |
| **Dermatological** | 19 (17.9%) | 1 (0.9%) |
| **Neurological** | 15 (14.2%) | 1 (0.9%) |
| **Haematological** | 23 (21.7%) | 14 (13.2%) |
| **Thyroid** | 2 (1.9%) | 0 |
| **Musculoskeletal** | 12 (11.3%) | 0 |
| **Genitourinary** | 4 (3.8%) | 1 (0.9%) |
| **Cardiological** | 7 (6.6%) | 3 (2.8%) |
| **Other** | 20 (18.9%) | 2 (1.9%) |

Supplementary Table 3. Crude analysis of prognostic factors associated with overall survival from second line initiation (n=106).

| Variable | HR | 95% CI | P-value |
|---|---|---|---|
| Systemic treatment adjuvant or neoadjuvant with radiotherapy or surgery before 1L | 0.699 | 0.255-1.913 | 0.485 |
| Palliative-intended radiotherapy before 2L | 1.058 | 0.610-1.835 | 0.842 |
| Radical-intended radiotherapy before 2L | 1.252 | 0.702-2.233 | 0.447 |
| 2L initiated for other causes than progression | 0.563 | 0.304-1.044 | 0.068 |
| 2L started within 30 days from end of 1L | 0.979 | 0.622-1.539 | 0.925 |
| 1L treatment days | 0.999 | 0.997-1.000 | 0.110 |
| Time from end of 1L to start of 2L | 0.999 | 0.997-1.001 | 0.279 |
| CRP before 2L | 1.005 | 0.998-1.012 | 0.180 |
| Albumin before 2L | 0.954 | 0.891-1.021 | 0.176 |
| eGFR before 2L | 1.000 | 0.985-1.015 | 0.990 |
| PS before 1L | | | |
| PS 0 | 0.680 | 0.375-1.233 | 0.204 |
| PS 1 | 0.825 | 0.532-1.279 | 0.391 |
| PS 2 | 1.795 | 1.111-2.901 | 0.017 |
| PS before 2L | | | |
| PS 0 | 0.637 | 0.327-1.239 | 0.184 |
| PS 1 | 0.736 | 0.472-1.149 | 0.177 |
| PS 2 | 1.733 | 1.104-2.722 | 0.017 |
| PS 3 n<5, not shown | | | |
| Target before 1L (total) | 1.001 | 0.996-1.006 | 0.665 |
| Largest target before 1L | 1.007 | 0.998-1.016 | 0.150 |
| Size reduction in 1L (total) | 1.005 | 0.999-1.011 | 0.091 |
| 1L size reduction of largest target | 1.005 | 0.999-1.010 | 0.125 |
| Target before 2L (total) | 1.003 | 0.997-1.008 | 0.316 |
| Largest target before 2L | 1.011 | 1.001-1.020 | 0.025 |
| Tumour doubling time between 1L and 2L | 1.000 | 1.000-1.000 | 0.951 |
| Tumour doubling time between 1L and 2L < 400 days | 0.931 | 0.558-1.554 | 0.785 |
| Sex (male) | 1.612 | 0.993-2.619 | 0.054 |
| BMI before 1L | 0.998 | 0.950-1.047 | 0.921 |
| 1L treatment | | | |
| Chemotherapy | 1.403 | 0.753-2.614 | 0.787 |
| Targeted therapy | 0.626 | 0.251-1.560 | 0.314 |
| Immunotherapy | 2.408 | 0.871-6.656 | 0.090 |

| | | | |
|---|---|---|---|
| Immunotherapy or immuno-chemotherapy | 0.849 | 0.389-1.853 | 0.681 |
| 1L dose reduced | 1.498 | 0.937-2.396 | 0.091 |
| Best 1L response (RECIST 1.1) | | | |
| CR | 0.043 | 0.001-3.302 | 0.155 |
| PR | 0.755 | 0.416-1.370 | 0.355 |
| SD | 1.165 | 0.748-1.814 | 0.500 |
| PD | 1.282 | 0.822-2.000 | 0.274 |
| Smoking status before 1L | | | |
| Non-smoker | 0.517 | 0.266-1.005 | 0.052 |
| Ex-smoker | 1.294 | 0.834-2.005 | 0.250 |
| Current smoker | 1.119 | 0.718-1.744 | 0.620 |
| Pack-years | 1.011 | 1.000-1.021 | 0.043 |
| Asbestos exposure | 1.793 | 1.038-3.097 | 0.036 |
| ISCO skill level | 1.050 | 0.782-1.411 | 0.745 |
| ISCO 1 | 0.958 | 0.503-1.826 | 0.897 |
| ISCO 2 | 1.098 | 0.691-1.745 | 0.693 |
| ISCO 3 | 0.641 | 0.325-1.264 | 0.199 |
| ISCO 4 | 1.699 | 0.807-3.574 | 0.163 |
| FEV1 before 1L | 0.994 | 0.979-1.010 | 0.471 |
| Charlson Comorbidity index set before 1L | | | |
| CCI 0-2 | 1.108 | 0.599-2.049 | 0.743 |
| CCI 3-4 | 0.749 | 0.374-1.500 | 0.414 |
| CCI 5-10 | 2.036 | 0.638-6.501 | 0.230 |
| Clinical Frailty scale before 1L | | | |
| 1-2 | 0.702 | 0.443-1.113 | 0.132 |
| 3-4 | 1.061 | 0.683-1.648 | 0.794 |
| 5-9 | 1.726 | 0.982-3.035 | 0.058 |
| CTCAE in 1L | | | |
| 0 | 0.937 | 0.548-1.602 | 0.812 |
| 1-2 | 0.732 | 0.472-1.137 | 0.165 |
| 3-4 | 1.547 | 0.970-2.468 | 0.067 |
| Primary TNM stage | | | |
| 1-2 | 0.917 | 0.441-1.907 | 0.817 |
| 3 | 0.926 | 0.559-1.536 | 0.767 |
| 4 | 1.103 | 0.697-1.744 | 0.676 |
| Histology | | | |
| Adenocarcinoma | 0.805 | 0.503-1.288 | 0.365 |
| Squamous | 1.253 | 0.764-2.057 | 0.372 |
| Other or unknown | 1.057 | 0.392-2.952 | 0.888 |
| Driver mutation positive (n=92) | 0.497 | 0.248-0.998 | 0.049 |
| PD-L1 status | | | |
| PD-L1 <1% | 1.282 | 0.752-2.184 | 0.361 |

| | | | |
|---|---|---|---|
| 1-49% | 0.793 | 0.448-1.403 | 0.425 |
| ≥50% | 0.960 | 0.529-1.742 | 0.893 |
| Guideline Based Treatment in 1L (optimal) | 0.791 | 0.403-1.551 | 0.495 |
| Distance to hospital km | 1.004 | 0.997-1.012 | 0.283 |
| PFS 1L Days | 0.997 | 0.996-0.999 | <0.001 |
| Age at 1L start | 1.015 | 0.988-1.042 | 0.275 |
| Age at 2L start | 1.013 | 0.986-1.039 | 0.348 |

All variables shown had less than 10% missing values; each estimate is from patients with complete data on that variable. Values are presented as hazard ratios (HRs) from crude (unadjusted) analyses of predictors associated with overall survival after second-line treatment. HR >1 indicates a higher hazard of death (worse overall survival), whereas HR <1 indicates a lower hazard (better overall survival). For continuous variables, estimates represent the effect per 1-unit increase in the variable as recorded. For categorical variables in which no reference category is explicitly shown, each category was analysed as an indicator variable. Abbreviations: 1L, first-line treatment; 2L, second-line treatment; HR, hazard ratio; CI, confidence interval; OS, overall survival; PFS, progression-free survival; PS, performance status; CRP, C-reactive protein; eGFR, estimated glomerular filtration rate; BMI, body mass index; RECIST, Response Evaluation Criteria in Solid Tumors; CR, complete response; PR, partial response; SD, stable disease; PD, progressive disease; ISCO, International Standard Classification of Occupations; FEV1, forced expiratory volume in 1 second; CCI, Charlson Comorbidity Index; CTCAE, Common Terminology Criteria for Adverse Events; TNM, tumour-node-metastasis; PD-L1, programmed death-ligand 1; CFS, Clinical Frailty Scale.

Supplementary Table 4. Multivariable-adjusted analysis of prognostic factors associated with overall survival during second-line therapy (n=106).

| Variable | Reference | aHR | 95% CI | p-value |
|---|---|---|---|---|
| **1L PFS (per day)** | — | 0.997 | 0.996–0.999 | <0.001 |
| **CTCAE grade 3–4 in 1L** | CTCAE grade 0–2 | 1.80 | 1.08–2.99 | 0.023 |
| **PS 2 before 1L** | PS 0–1 | 1.80 | 1.03–3.14 | 0.038 |
| **Chemotherapy in 1L** | No chemotherapy | 1.04 | 0.51–2.11 | 0.908 |
| **Pack-years** | — | 1.001 | 0.988–1.014 | 0.886 |
| **Sex, male** | Female | 0.97 | 0.57–1.64 | 0.911 |

Values are adjusted hazard ratios (aHRs) from a multivariable Cox proportional hazards regression model. An aHR >1 indicates higher hazard of death (worse OS); aHR <1 indicates lower hazard. Continuous variables represent the effect per 1-unit increase. Non-significant variables are retained as pre-specified by clinical relevance. Abbreviations: aHR, adjusted hazard ratio; CI, confidence interval; CTCAE, Common Terminology Criteria for Adverse Events; OS, overall survival; PFS, progression-free survival; PS, performance status; 1L, first-line therapy. No chemotherapy denotes first-line regimens other than chemotherapy alone (immunotherapy, chemoimmunotherapy, or targeted therapy).

Supplementary Table 5. Absolute differences in 12-month overall survival across key prognostic subgroups

| Variable | Comparison | OS ≥12 month (%) | Absolute difference (percentage points) |
|---|---|---|---|
| CTCAE 1L toxicity | CTCAE 0–2 vs 3–4 | 69.1% vs 39.2% | 29.9 |
| Frailty (CFS) | CFS 1–2 vs ≥3 | 66.7% vs 52.3% | 14.4 |
| Performance status before 1L | PS 0 vs PS 2 | 68.4% vs 30.8% | 37.6 |
| | PS 1 vs PS 2 | 60.7% vs 30.8% | 29.9 |
| | PS 0 vs PS 1 | 68.4% vs 60.7% | 7.7 |

Absolute differences represent descriptive differences in the proportion of patients achieving OS ≥12 months across clinical subgroups. These comparisons are observational and prognostic in nature and should not be interpreted as treatment effects. 1L = first-line treatment; CFS = Clinical Frailty Scale; CTCAE = Common Terminology Criteria for Adverse Events; OS = overall survival; PS = performance status.

Supplementary Table 6. Crude analysis of prognostic factors associated with progression-free survival during second-line therapy (n=106).

| Variable | HR | 95% CI | P-value |
|---|---|---|---|
| Systemic treatment adjuvant or neoadjuvant with radiotherapy or surgery before 1L | 0.797 | 0.322-1.970 | 0.623 |
| Palliative-intended radiotherapy before 2L | 0.891 | 0.536-1.478 | 0.654 |
| Radical-intended radiotherapy before 2L | 1.083 | 0.631-1.860 | 0.772 |
| 2L initiated for other causes than progression | 0.628 | 0.363-1.086 | 0.096 |
| 2L started within 30 days from end of 1L | 0.728 | 0.479-1.108 | 0.138 |
| 1L Treatment days | 0.999 | 0.998-1.001 | 0.302 |
| Time from end of 1L to start of 2L | 1.000 | 0.998-1.001 | 0.697 |
| CRP before 2L | 1.003 | 0.997-1.010 | 0.338 |
| Albumin before 2L | 0.977 | 0.929-1.028 | 0.369 |
| eGFR before 2L | 0.999 | 0.986-1.012 | 0.877 |
| PS before 1L | | | |
| PS 0 | 0.540 | 0.305-0.955 | 0.034 |
| PS 1 | 0.998 | 0.661-1.505 | 0.992 |
| PS 2 | 1.785 | 1.122-2.840 | 0.014 |
| PS before 2L | | | |

| | | | |
|---|---|---|---|
| PS 0 | 0.852 | 0.463-1.569 | 0.608 |
| PS 1 | 0.734 | 0.488-1.104 | 0.137 |
| PS 2 | 1.488 | 0.975-2.272 | 0.065 |
| PS 3 n<5, not shown | | | |
| Target before 1L (total) | 1.001 | 0.996-1.006 | 0.656 |
| Largest target before 1L | 1.003 | 0.994-1.011 | 0.514 |
| Size reduction in 1L (total) | 1.002 | 0.998-1.007 | 0.303 |
| 1L size reduction of largest target | 1.002 | 0.997-1.006 | 0.426 |
| Target before 2L (total) | 1.002 | 0.997-1.007 | 0.468 |
| Largest target before 2L | 1.003 | 0.995-1.012 | 0.441 |
| Tumour doubling time between 1L and 2L | 1.000 | 1.000-1.000 | 0.837 |
| Tumour doubling time between 1L and 2L < 400 days | 0.987 | 0.616-1.583 | 0.958 |
| Sex (male) | 1.350 | 0.876-2.079 | 0.174 |
| BMI before 1L | 1.003 | 0.957-1.051 | 0.905 |
| 1L treatment | | | |
| Chemotherapy | 1.996 | 1.144-3.482 | 0.015 |
| Targeted therapy | 0.476 | 0.208-1.092 | 0.080 |
| Immunotherapy | 1.635 | 0.596-4.489 | 0.340 |
| Immunotherapy or immuno-chemotherapy | 0.609 | 0.306-1.215 | 0.159 |
| 1L Treatment reduced | 1.632 | 1.056-2.522 | 0.027 |
| Best 1L response (RECIST 1.1) | | | |
| CR | 0.239 | 0.058-0.987 | 0.048 |
| PR | 0.893 | 0.527-1.515 | 0.676 |
| SD | 0.926 | 0.604-1.420 | 0.724 |
| PD | 1.509 | 0.990-2.299 | 0.056 |
| Smoking status before 1L | | | |
| Non-smoker | 0.457 | 0.248-0.844 | 0.012 |
| Ex-smoker | 1.391 | 0.921-2.101 | 0.116 |
| Current smoker | 1.154 | 0.760-1.754 | 0.501 |
| Pack-years | 1.013 | 1.004-1.023 | 0.005 |
| Asbestos exposure | 1.584 | 0.951-2.637 | 0.077 |
| ISCO skill level | 0.912 | 0.689-1.209 | 0.523 |
| ISCO 1 | 0.925 | 0.512-1.670 | 0.796 |
| ISCO 2 | 1.560 | 0.993-2.452 | 0.054 |
| ISCO 3 | 0.342 | 0.161-0.728 | 0.005 |
| ISCO 4 | 1.531 | 0.734-3.197 | 0.256 |
| FEV1 before 1L | 0.994 | 0.980-1.008 | 0.405 |
| Charlson Comorbidity index set before 1L | | | |
| CCI 0-2 | 0.832 | 0.483-1.432 | 0.506 |

| | | | |
|---|---|---|---|
| CCI 3-4 | 1.055 | 0.584-1.904 | 0.859 |
| CCI 5-10 | 2.364 | 0.735-7.606 | 0.149 |
| Clinical Frailty scale before 1L | | | |
| 1-2 | 0.667 | 0.438-1.015 | 0.059 |
| 3-4 | 1.259 | 0.838-1.893 | 0.268 |
| 5-9 | 1.430 | 0.818-2.500 | 0.208 |
| CTCAE in 1L | | | |
| 0 | 0.673 | 0.405-1.118 | 0.126 |
| 1-2 | 0.844 | 0.561-1.268 | 0.413 |
| 3-4 | 1.805 | 1.163-2.799 | 0.008 |
| Primary TNM stage | | | |
| 1-2 | 1.005 | 0.520-1.945 | 0.987 |
| 3 | 0.882 | 0.546-1.427 | 0.610 |
| 4 | 1.104 | 0.719-1.694 | 0.651 |
| Histology | | | |
| Adenocarcinoma | 0.875 | 0.567-1.349 | 0.545 |
| Squamous | 1.144 | 0.726-1.801 | 0.562 |
| Other or unknown | 1.056 | 0.422-2.639 | 0.907 |
| Driver mutation positive (n=92) | 0.394 | 0.203-0.765 | 0.006 |
| PD-L1 status | | | |
| PD-L1 <1% | 1.262 | 0.770-2.069 | 0.357 |
| 1-49% | 0.803 | 0.476-1.356 | 0.412 |
| ≥50% | 0.968 | 0.553-1.694 | 0.910 |
| Guideline Based Treatment in 1L (optimal) | 0.567 | 0.287-1.119 | 0.102 |
| Distance to hospital km | 1.003 | 0.996-1.011 | 0.344 |
| PFS 1L Days | 0.999 | 0.998-1.000 | 0.003 |
| Age at 1L start | 1.005 | 0.981-1.028 | 0.705 |
| Age at 2L start | 1.003 | 0.981-1.027 | 0.770 |

All variables shown had less than 10% missing values; each estimate is from patients with complete data on that variable. Values are presented as hazard ratios (HRs) from crude (unadjusted) analyses of predictors associated with progression-free survival after second-line treatment. HR >1 indicates a higher hazard of progression or death (worse progression-free survival), whereas HR <1 indicates a lower hazard (better progression-free survival). For continuous variables, estimates represent the effect per 1-unit increase in the variable as recorded. For categorical variables in which no reference category is explicitly shown, each category was analysed as an indicator variable. Abbreviations: 1L, first-line treatment; 2L, second-line treatment; HR, hazard ratio; CI, confidence interval; PFS, progression-free survival; PS, performance status; CRP, C-reactive protein; eGFR, estimated glomerular filtration rate; BMI, body mass index; RECIST, Response Evaluation Criteria in Solid Tumors; CR, complete response; PR, partial response; SD, stable disease; PD, progressive disease; ISCO, International Standard Classification of Occupations; FEV1, forced expiratory volume in 1 second; CCI, Charlson Comorbidity Index; CTCAE, Common Terminology Criteria for Adverse Events; TNM, tumour-node-metastasis; PD-L1, programmed death-ligand 1; CFS, Clinical Frailty Scale.

Supplementary Table 7. Multivariable-adjusted analysis of prognostic factors associated with progression-free survival during second-line therapy (n=106).

| Variable | Reference | aHR | 95% CI | p-value |
|---|---|---|---|---|
| Chemotherapy in 1L | No chemotherapy | 1.600 | 0.860-2.978 | 0.138 |
| PFS 1L, Days | | 0.998 | 0.998-0.999 | <0.001 |
| CTCAE 3-4 1L | CTCAE 0-2 | 2.333 | 1.414-3.850 | <0.001 |
| PS 2 1L | PS 0-1 | 1.648 | 0.915-2.968 | 0.096 |
| Packyears | | 1.010 | 0.999-1.021 | 0.084 |
| Sex, male | female | 0.860 | 0.532-1.390 | 0.537 |

Values are presented as adjusted hazard ratios (aHRs) from a multivariable Cox proportional hazards regression model of clinically relevant prognostic factors associated

with progression-free survival after second-line treatment initiation. Reference categories for categorical predictors are shown in the second column. An aHR >1 indicates a higher hazard of progression or death (worse PFS), whereas an aHR <1 indicates a lower hazard (better PFS). For continuous variables, estimates represent the effect per 1-unit increase in the variable as recorded. Variables not reaching statistical significance are retained in the model as they were selected a priori based on clinical relevance. Abbreviations: 1L, first-line treatment; aHR, adjusted hazard ratio; CI, confidence interval; PFS, progression-free survival; PS, performance status; CTCAE, Common Terminology Criteria for Adverse Events. No chemotherapy denotes first-line regimens other than chemotherapy alone (immunotherapy, chemoimmunotherapy, or targeted therapy).

Supplementary Table 8. Crude analysis of factors associated with progressive disease as best radiologic response during second-line therapy (n=106).

| Variable | OR | 95% CI | P-value |
|---|---|---|---|
| Systemic treatment adjuvant or neoadjuvant with radiotherapy or surgery before 1L | 0.67 | 0.124-3.631 | 0.643 |
| Palliative-intended radiotherapy before 2L | 1.246 | 0.477-3.254 | 0.653 |
| Radical-intended radiotherapy before 2L | 0.475 | 0.143-1.574 | 0.223 |

| | | | |
|---|---|---|---|
| 2L initiated for other causes than progression | 0.755 | 0.262-2.18 | 0.604 |
| 2L started within 30 days from end of 1L | 1.116 | 0.486-2.566 | 0.796 |
| 1L Treatment days | 0.998 | 0.995-1.001 | 0.148 |
| Time from end of 1L to start of 2L | 0.997 | 0.994-1.001 | 0.128 |
| CRP before 2L | 1.001 | 0.988-1.015 | 0.846 |
| Albumin before 2L | 1.002 | 0.924-1.087 | 0.955 |
| eGFR before 2L | 1.010 | 0.985-1.035 | 0.443 |
| PS before 1L | | | |
| PS 0 | 0.557 | 0.184-1.686 | 0.300 |
| PS 1 | 1.097 | 0.493-2.443 | 0.821 |
| PS 2 | 1.362 | 0.552-3.362 | 0.503 |
| PS before 2L | | | |
| PS 0 | 1.066 | 0.323-3.522 | 0.916 |
| PS 1 | 0.841 | 0.381-1.858 | 0.669 |
| PS 2 | 1.047 | 0.458-2.295 | 0.913 |
| PS 3 n<5, not shown | | | |
| Target before 1L (total) | 1.004 | 0.994-1.013 | 0.464 |
| Largest target before 1L | 1.008 | 0.992-1.024 | 0.325 |
| Size reduction in 1L (total) | 1.006 | 0.995-1.017 | 0.259 |
| 1L size reduction of largest target | 1.006 | 0.995-1.016 | 0.281 |
| Target before 2L (total) | 1.004 | 0.994-1.013 | 0.480 |
| Largest target before 2L | 1.008 | 0.991-1.026 | 0.377 |
| Tumour doubling time between first and second line | 1.000 | 1.000-1.000 | 0.424 |
| Tumour doubling time between 1L and 2L < 400 days | 0.597 | 0.235-1.517 | 0.278 |
| Sex (male) | 1.427 | 0.617-3.302 | 0.406 |
| BMI before 1L | 0.977 | 0.888-1.076 | 0.643 |
| 1L treatment | | | |
| Chemotherapy | 3.464 | 1.083-11.081 | 0.036 |
| Targeted therapy | 0.170 | 0.021-1.393 | 0.099 |
| Immunotherapy | 1.757 | 0.237-12.995 | 0.581 |
| Immunotherapy or immuno-chemotherapy | 0.475 | 0.122-1.843 | 0.282 |
| 1L Treatment reduced | 1.816 | 0.799-4.128 | 0.154 |
| Best 1L response (RECIST 1.1) | | | |
| CR | 0.561 | 0.056-5.591 | 0.623 |
| PR | 0.909 | 0.328-2.514 | 0.854 |
| SD | 0.840 | 0.366-1.926 | 0.680 |
| PD | 1.352 | 0.608-3.004 | 0.460 |
| Smoking status before 1L | | | |
| Non-smoker | 0.833 | 0.286-2.431 | 0.739 |

| | | | |
|---|---|---|---|
| Ex-smoker | 1.076 | 0.485-2.390 | 0.857 |
| Current smoker | 1.031 | 0.462-2.301 | 0.941 |
| Pack-years | 1.011 | 0.993-1.029 | 0.231 |
| Asbestos exposure | 1.527 | 0.570-4.095 | 0.400 |
| ISCO skill level | 1.317 | 0.780-2.222 | 0.303 |
| ISCO 1 | 0.753 | 0.240-2.365 | 0.627 |
| ISCO 2 | 0.986 | 0.428-2.272 | 0.973 |
| ISCO 3 | 0.655 | 0.190-2.257 | 0.502 |
| ISCO 4 | 3.177 | 0.713-14.152 | 0.129 |
| FEV1 before 1L | 0.982 | 0.954-1.010 | 0.209 |
| Charlson Comorbidity index set before 1L | | | |
| CCI 0-2 | 1.080 | 0.366-3.193 | 0.889 |
| CCI 3-4 | 0.651 | 0.190-2.237 | 0.496 |
| CCI 5-10 | 3.568 | 0.313-40.685 | 0.306 |
| Clinical Frailty scale before 1L | | | |
| 1-2 | 0.405 | 0.174-0.943 | 0.036 |
| 3-4 | 2.576 | 1.145-5.791 | 0.022 |
| 5-9 | 0.838 | 0.264-2.659 | 0.765 |
| CTCAE in 1L | | | |
| 0 | 0.346 | 0.118-1.012 | 0.053 |
| 1-2 | 0.761 | 0.343-1.690 | 0.502 |
| 3-4 | 2.971 | 1.267-6.968 | 0.012 |
| Primary TNM stage | | | |
| 1-2 | 0.980 | 0.268-3.585 | 0.975 |
| 3 | 0.553 | 0.208-1.465 | 0.233 |
| 4 | 1.614 | 0.688-3.789 | 0.271 |
| Histology | | | |
| Adenocarcinoma | 0.851 | 0.365-1.985 | 0.709 |
| Squamous | 1.155 | 0.475-2.809 | 0.750 |
| Other or unknown | 1.153 | 0.184-7.220 | 0.879 |
| Driver mutation positive (n=92) | 0.346 | 0.092-1.301 | 0.116 |
| PD-L1 status | | | |
| PD-L1 <1% | 1.179 | 0.446-3.114 | 0.740 |
| 1-49% | 0.515 | 0.175-1.519 | 0.229 |
| ≥50% | 1.668 | 0.570-4.885 | 0.350 |
| Guideline Based Treatment in 1L (optimal) | 1.480 | 0.351-6.239 | 0.594 |
| Distance to hospital km | 1.002 | 0.989-1.017 | 0.732 |
| PFS 1L Days | 0.997 | 0.994-0.999 | 0.018 |
| Age at 1L start | 1.012 | 0.967-1.060 | 0.605 |
| Age at 2L start | 1.009 | 0.964-1.055 | 0.701 |

All variables shown had less than 10% missing values; each estimate is from patients with complete data on that variable. Values are presented as odds ratios (ORs) from crude (unadjusted) analyses of second-line treatment radiologic response in relation to progressive disease. OR >1 indicates higher odds of progressive disease relative to non-progressive disease (stable disease, partial response, or complete response), whereas OR <1 indicates lower odds of progressive disease. For continuous variables, estimates represent the effect per 1-unit increase in the variable as recorded. For categorical variables in which no reference category is explicitly shown, each category was analysed as an indicator variable. Abbreviations: 1L, first-line treatment; 2L, second-line treatment; OR, odds ratio; CI, confidence interval; PS, performance status; CRP, C-reactive protein; eGFR, estimated glomerular filtration rate; BMI, body mass index; RECIST, Response Evaluation Criteria in Solid Tumors; CR, complete response; PR, partial response; SD, stable disease; PD, progressive disease; ISCO, International Standard Classification of Occupations; FEV1, forced expiratory volume in 1 second; CCI, Charlson Comorbidity Index; CTCAE, Common Terminology Criteria for Adverse Events; TNM, tumour-node-metastasis; PD-L1, programmed death-ligand 1; PFS, progression-free survival; CFS, Clinical Frailty Scale.

Supplementary Table 9. Multivariable-adjusted analysis of prognostic factors associated with progressive disease as best radiologic response during second-line therapy (n=92).

| Variable | Reference | aOR | 95% CI | p-value |
|---|---|---|---|---|
| Chemotherapy in 1L | No chemotherapy | 3.514 | 0.857-14.404 | 0.081 |
| PFS 1L, Days | | 0.996 | 0.993-0.999 | 0.011 |
| CTCAE 3-4 1L | CTCAE 0-2 | 2.807 | 1.026-7.682 | 0.044 |
| Driver mutation positivity | No | 1.483 | 0.258-8.510 | 0.659 |
| CFS 1-2 | CFS 3-9 | 0.374 | 0.141-0.996 | 0.049 |
| Sex, male | female | 1.086 | 0.376-3.134 | 0.879 |

Fourteen patients with untested driver mutation status were excluded from this model; all other variables were complete, giving n=92. Values are presented as adjusted odds ratios (aORs) from a multivariable logistic regression model in which progressive disease at best radiologic response during second-line treatment was compared with stable disease/partial response/complete response. Reference categories for predictors are shown in the second column. An aOR >1 indicates higher odds of progressive disease, whereas an aOR <1 indicates lower odds of progressive disease. For continuous variables, estimates represent the effect per 1-unit increase in the variable as recorded. Abbreviations: 1L, first-line treatment; aOR, adjusted odds ratio; CI, confidence interval; PFS, progression-free survival; CTCAE, Common Terminology Criteria for Adverse Events; CFS, Clinical Frailty Scale. No chemotherapy denotes first-line regimens other than chemotherapy alone (immunotherapy, chemoimmunotherapy, or targeted therapy).
